# Supplementary material for: Diversifying Evolution of the Ubiquitin-26S Proteasome System in Brassicaceae and Poaceae
Source: Int J Mol Sci. 2019 Jun 30;20(13):3226. doi: 10.3390/ijms20133226 (PMC6651606; doi:10.3390/ijms20133226)
Supplement: Supplementary file 1 [file ijms-20-03226-s001.zip › supplementary_Files/Supplemental_Tables/Table S1.docx]

**Table S1.** List of Pfam families for annotating the UPS members in this work

| UPS Family | Pfam Family | Pfam ID |
| --- | --- | --- |
| APC | ANAPC1 | PF12859 |
| APC | ANAPC2 | PF08672 |
| APC | ANAPC3 | PF12895 |
| APC | ANAPC4 | PF12896 |
| APC | ANAPC5 | PF12862 |
| APC | ANAPC9 | PF12856 |
| APC | ANAPC10 | PF03256 |
| APC | ANAPC15 | PF15243 |
| APC | ANAPC16 | PF17256 |
| APC | ANAPC_CDC26 | PF10471 |
| BTB | BTB | PF00651 |
| BTB | BTB_2 | PF02214 |
| BTB | BTB_3 | PF16017 |
| CP | Proteasome | PF00227 |
| CP | PA28_beta | PF02252 |
| CP | PA28_alpha | PF02251 |
| CP | Hydrophob_seed | PF14547 |
| Cullin | Cullin | PF00888 |
| Cullin | Cullin_Nedd8 | PF10557 |
| E1 | UBA_e1_thiolCys | PF10585 |
| E1 | E1_UFD | PF09358 |
| E2 | UQ_con | PF00179 |
| F-box | F-box | PF00646 |
| F-box | F-box-like | PF12937 |
| HECT | HECT | PF00632 |
| RING | RINGv | PF12906 |
| RING | zf-RING_5 | PF14634 |
| RING | zf-rbx1 | PF12678 |
| RING | zf-C3HC4 | PF00097 |
| RING | zf-RING_UBOX | PF13445 |
| RING | zf-RING_4 | PF14570 |
| RING | zf-ANAPC11 | PF12861 |
| RING | zf-RING_2 | PF13639 |
| RING | zf-RING_11 | PF17123 |
| RING | zf-C3HC4_3 | PF13920 |
| RING | zf-C3HC4_2 | PF13923 |
| RING | FANCL_C | PF11793 |
| RP | AAA_lid_3 | PF17862 |
| RP | AAA | PF00004 |
| RP | CSN8_PSD8_EIF3K | PF10075 |
| RP | HEAT_2 | PF13646 |
| RP | JAB | PF01398 |
| RP | MitMem_reg | PF13012 |
| RP | PC_rep | PF01851 |
| RP | PCI | PF01399 |
| RP | Prot_ATP_ID_OB | PF16450 |
| RP | Proteasom_Rpn13 | PF04683 |
| RP | RPN1_C | PF18051 |
| RP | RPN1_RPN2_N | PF17781 |
| RP | RPN2_C | PF18004 |
| RP | RPN3_C | PF08375 |
| RP | RPN5_C | PF18098 |
| RP | RPN6_C_helix | PF18503 |
| RP | RPN6_N | PF18055 |
| RP | RPN7 | PF10602 |
| RP | UIM | PF02809 |
| RP | VWA_2 | PF13519 |
| RP | YfdX | PF10938 |
| Skp1 | Skp1 | PF01466 |
