# Supplementary material for: Diversifying Evolution of the Ubiquitin-26S Proteasome System in Brassicaceae and Poaceae
Source: Int J Mol Sci. 2019 Jun 30;20(13):3226. doi: 10.3390/ijms20133226 (PMC6651606; doi:10.3390/ijms20133226)
Supplement: Supplementary file 1 [file ijms-20-03226-s001.zip › supplementary_Files/Supplemental_Tables/Table S2.docx]

**Table S2.** Data resources of 14 genomes analyzed in this work

| **Group** | **Species Name** | **Abbreviation** | **Version** | **Data Source** |
| --- | --- | --- | --- | --- |
| *Angiosperm* | *Amborella trichopoda* | *Atr* | Amborella trichopoda v1.0 | Phytozome 12 |
| *Brassicaceae* | *Arabidopsis halleri* | *Aha* | Arabidopsis halleri v1.1 | Phytozome 12 |
| *Brassicaceae* | *Arabidopsis lyrata* | *Aly* | Arabidopsis lyrata v2.1 | Phytozome 12 |
| *Brassicaceae* | *Arabidopsis thaliana* | *Ath* | TAIR10 | Phytozome 12 |
| *Brassicaceae* | *Brachypodium distachyon* | *Bdi* | Brachypodium distachyon Bd21-3 v1.1 | Phytozome 12 |
| *Brassicaceae* | *Brassica rapa* | *Bra* | Brassica rapa FPsc v1.3 | Phytozome 12 |
| *Brassicaceae* | *Boechera stricta* | *Bst* | Boechera stricta v1.2 | Phytozome 12 |
| *Brassicaceae* | *Capsella rubella* | *Cru* | Capsella rubella v1.0 | Phytozome 12 |
| *Poaceae* | *Leersia perrieri* | *Lpe* | Lperr_V1.4 | Ensembl |
| *Poaceae* | *Oryza brachyantha* | *Obr* | Oryza_brachyantha.v1.4b | Ensembl |
| *Poaceae* | *Oryza punctata* | *Opu* | Oryza_punctata_v1.2 | Ensembl |
| *Poaceae* | *Oryza sativa* | *Osa* | Oryza sativa v7_JGI | Phytozome 12 |
| *Poaceae* | *Sorghum bicolor* | *Sbi* | Sorghum bicolor v3.1.1 | Phytozome 12 |
| *Poazeae* | *Zea mays* | *Zma* | Zea_mays.B73_RefGen_v4 | Ensembl |
