# Supplementary material for: Diversifying Evolution of the Ubiquitin-26S Proteasome System in Brassicaceae and Poaceae
Source: Int J Mol Sci. 2019 Jun 30;20(13):3226. doi: 10.3390/ijms20133226 (PMC6651606; doi:10.3390/ijms20133226)
Supplement: Supplementary file 1 [file ijms-20-03226-s001.zip › supplementary_Files/Supplemental_Tables/Table S3.docx]

**Table S3.** Sizes comparison of the UPS in 14 genomes

| **Species** | ***Aly*** | ***Aha*** | ***Ath*** | ***Cru*** | ***Bst*** | ***Bra*** | ***Osa*** | ***Opu*** | ***Obr*** | ***Lpe*** | ***Bdi*** | ***Sbi*** | ***Zma*** | ***Atr*** |
| --- | --- | --- | --- | --- | --- | --- | --- | --- | --- | --- | --- | --- | --- | --- |
| Prior  Annotation | 1713 | 1386 | 1415 | 1692 | 1173 | 2062 | 1519 | 1262 | 853 | 1243 | 1655 | 1495 | 1298 | 626 |
| New  Pep | 71 | 151 | 15 | 31 | 49 | 77 | 63 | 21 | 33 | 23 | 47 | 51 | 109 | 104 |
| New  Pseudo | 105 | 85 | 30 | 36 | 127 | 65 | 70 | 41 | 27 | 35 | 63 | 64 | 199 | 115 |
| Total | 1889 | 1622 | 1460 | 1759 | 1349 | 2204 | 1652 | 1324 | 913 | 1301 | 1765 | 1610 | 1606 | 845 |
| New  Discovery  Rate  (% of total) | 9.3 | 14.5 | 3.1 | 3.8 | 13.0 | 6.4 | 8.1 | 4.7 | 6.6 | 4.5 | 6.2 | 7.1 | 19.2 | 25.9 |
