# Supplementary material for: Diversifying Evolution of the Ubiquitin-26S Proteasome System in Brassicaceae and Poaceae
Source: Int J Mol Sci. 2019 Jun 30;20(13):3226. doi: 10.3390/ijms20133226 (PMC6651606; doi:10.3390/ijms20133226)
Supplement: Supplementary file 1 [file ijms-20-03226-s001.zip › supplementary_Files/Supplemental_Tables/Table S4.docx]

**Table S4.** Number of protein sequences identified in 11 UPS families in 14 genomes

| **Species** | ***APC*** | ***BTB*** | ***Cullin*** | ***E1*** | ***E2*** | ***FBX*** | ***HECT*** | ***CP*** | ***RP*** | ***Skp1*** | ***RING*** | **Sum** |
| --- | --- | --- | --- | --- | --- | --- | --- | --- | --- | --- | --- | --- |
| *Aha* | 14 | 63 | 5 | 2 | 44 | 850 | 9 | 27 | 29 | 14 | 480 | 1537 |
| *Aly* | 18 | 77 | 4 | 3 | 53 | 989 | 10 | 34 | 33 | 21 | 542 | 1784 |
| *Ath* | 16 | 64 | 6 | 2 | 50 | 697 | 7 | 24 | 27 | 21 | 516 | 1430 |
| *Atr* | 16 | 56 | 4 | 1 | 40 | 230 | 7 | 25 | 23 | 14 | 314 | 730 |
| *Bdi* | 10 | 178 | 7 | 4 | 56 | 813 | 10 | 26 | 31 | 13 | 554 | 1702 |
| *Bra* | 21 | 97 | 12 | 4 | 99 | 975 | 10 | 44 | 49 | 26 | 802 | 2139 |
| *Bst* | 18 | 75 | 5 | 2 | 62 | 505 | 9 | 28 | 27 | 18 | 473 | 1222 |
| *Cru* | 12 | 69 | 5 | 2 | 50 | 970 | 8 | 26 | 28 | 26 | 527 | 1723 |
| *Lpe* | 15 | 110 | 6 | 2 | 46 | 542 | 9 | 26 | 28 | 22 | 460 | 1266 |
| *Obr* | 11 | 78 | 6 | 3 | 48 | 264 | 12 | 26 | 23 | 24 | 391 | 886 |
| *Opu* | 13 | 111 | 7 | 4 | 46 | 535 | 8 | 25 | 27 | 22 | 485 | 1283 |
| *Osa* | 13 | 156 | 8 | 4 | 51 | 732 | 8 | 23 | 28 | 27 | 532 | 1582 |
| *Sbi* | 10 | 158 | 6 | 4 | 54 | 678 | 9 | 25 | 25 | 14 | 563 | 1546 |
| *Zma* | 20 | 95 | 8 | 5 | 97 | 331 | 20 | 79 | 35 | 18 | 699 | 1407 |
| Sum | 207 | 1387 | 89 | 42 | 796 | 9111 | 136 | 438 | 413 | 280 | 7338 | 20237 |
