# Supplementary material for: Diversifying Evolution of the Ubiquitin-26S Proteasome System in Brassicaceae and Poaceae
Source: Int J Mol Sci. 2019 Jun 30;20(13):3226. doi: 10.3390/ijms20133226 (PMC6651606; doi:10.3390/ijms20133226)
Supplement: Supplementary file 1 [file ijms-20-03226-s001.zip › supplementary_Files/Supplemental_Tables/Table S5.docx]

**Table S5.** Correlation assay of *K_a_/K_s_, K_a_,* and *K_s_* values of with age ranks

| Family | Species | *K_a_/K_s_* | | *K_a_* | | *K_s_* | |
| --- | --- | --- | --- | --- | --- | --- | --- |
|  |  | ρ | P-value | ρ | P-value | ρ | P-value |
| *FBX* | *Aly* | -1 | 0 | 1 | 0 | 1 | 0 |
|  | *Osa* | -0.2 | 0.8 | 0.8 | 0.2 | 0.8 | 0.2 |
| *BTB* | *Aly* | -0.8 | 0.2 | 0.4 | 0.6 | 0.8 | 0.2 |
|  | *Osa* | -0.4 | 0.6 | -0.8 | 0.2 | -0.4 | 0.6 |
| *RING* | *Aly* | -1 | 0 | -1 | 0 | -0.4 | 0.6 |
|  | *Osa* | -1 | 0 | -1 | 0 | -0.8 | 0.2 |
| *Skp1* | *Aly* | -1 | 0 | -0.8 | 0.2 | 0.4 | 0.6 |
|  | *Osa* | N/A | N/A | N/A | N/A | N/A | N/A |

* Correlation assays between the values of each category and age ranks from 7 to 10 as shown in Figure 4 were performed using a Spearman’s method.
