# Supplementary figures and images for: Diversifying Evolution of the Ubiquitin-26S Proteasome System in Brassicaceae and Poaceae
Source: Int J Mol Sci. 2019 Jun 30;20(13):3226. doi: 10.3390/ijms20133226 (PMC6651606; doi:10.3390/ijms20133226)

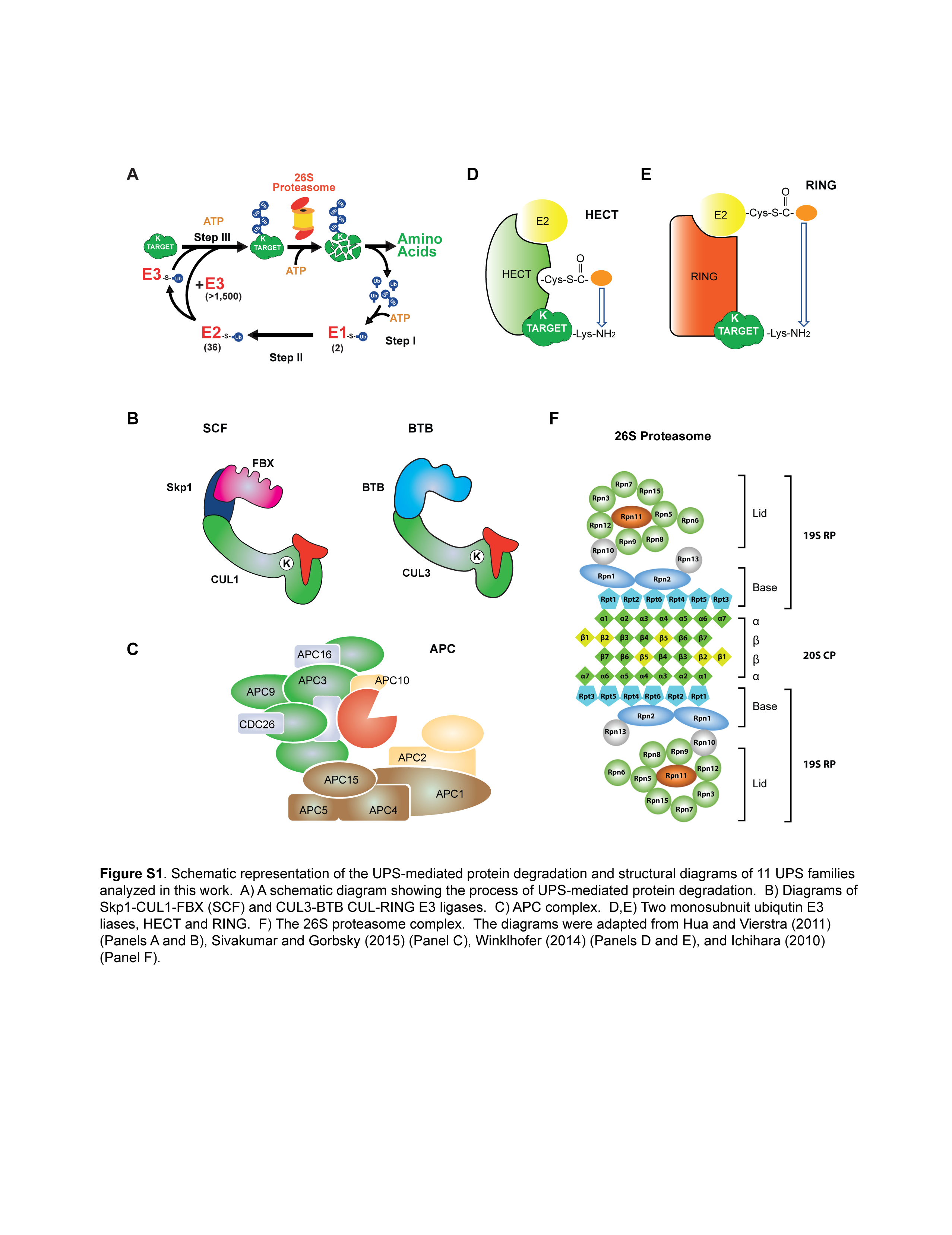

Supplement: Supplementary file 1 [file ijms-20-03226-s001.zip › supplementary_Files/Supplemental_Figures_tiff/Figure S1.tif]

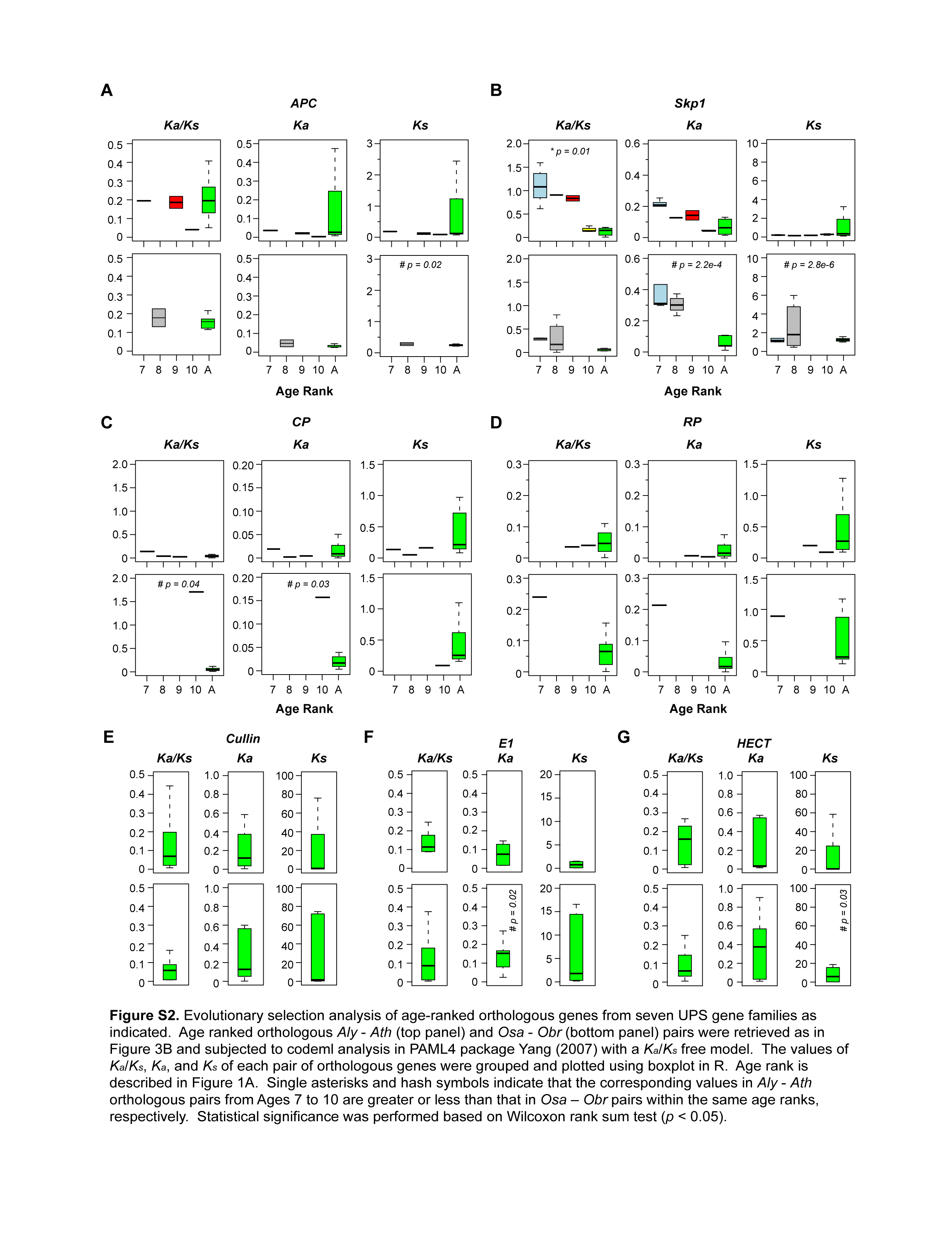

Supplement: Supplementary file 1 [file ijms-20-03226-s001.zip › supplementary_Files/Supplemental_Figures_tiff/Figure S2.tif]

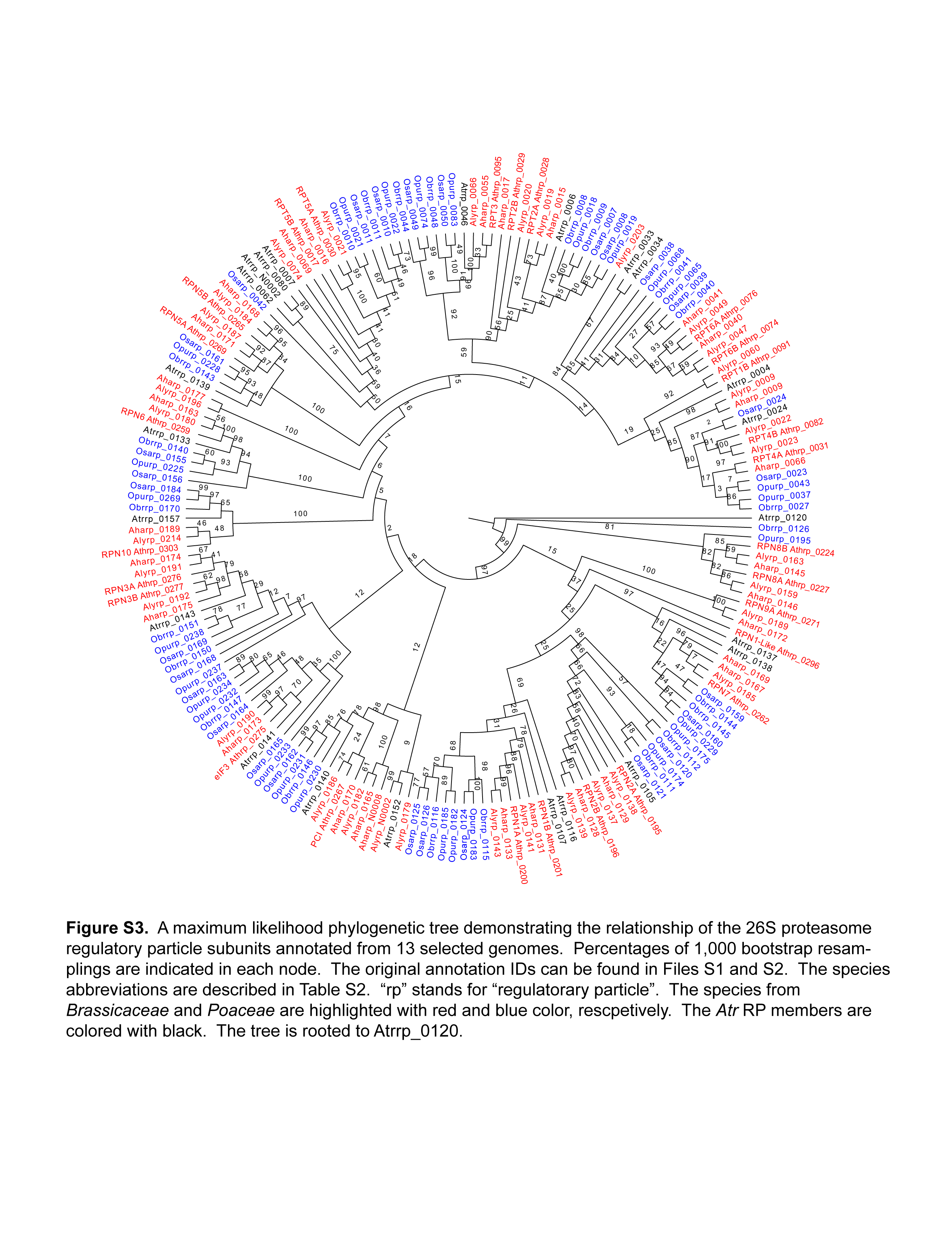

Supplement: Supplementary file 1 [file ijms-20-03226-s001.zip › supplementary_Files/Supplemental_Figures_tiff/Figure S3.tif]

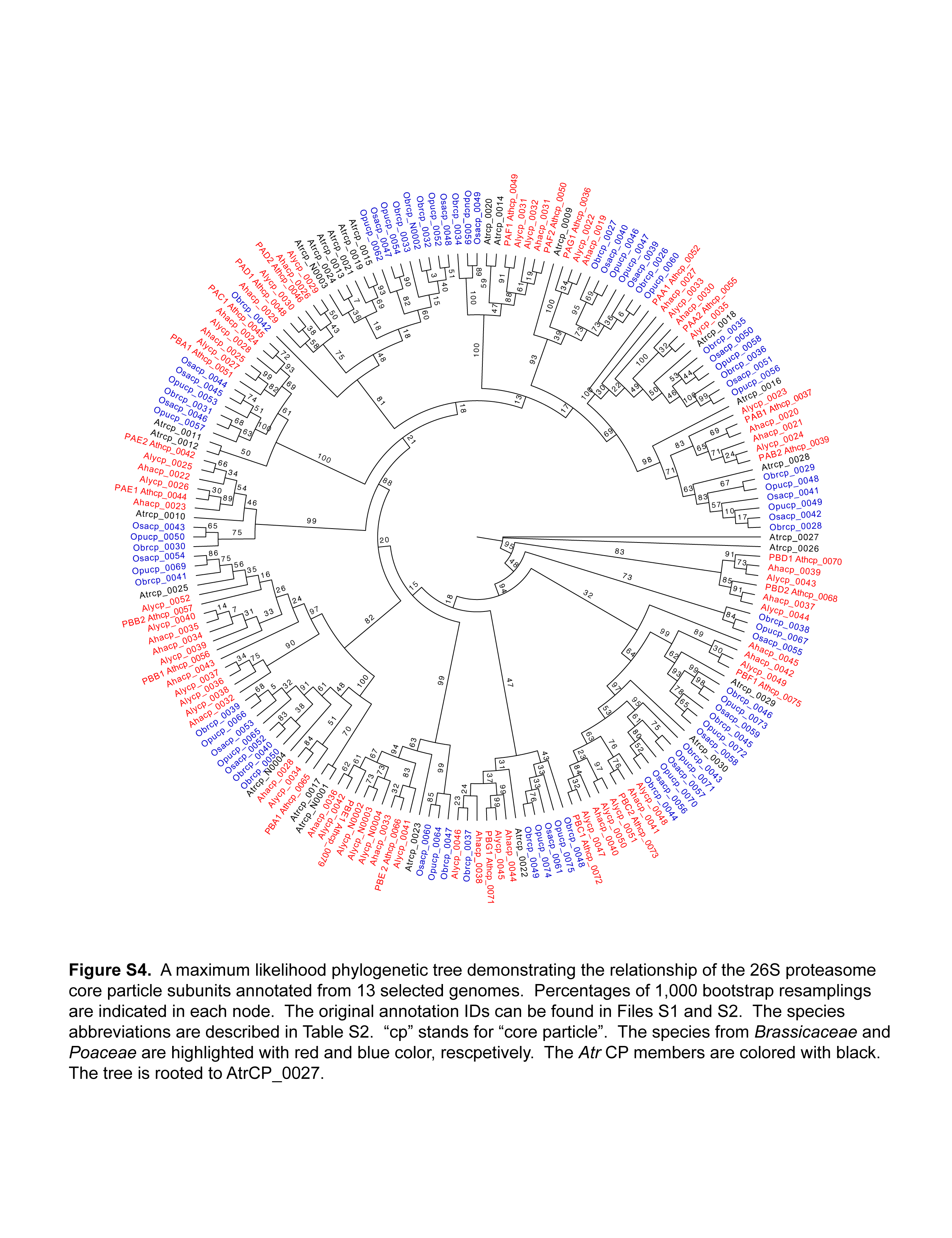

Supplement: Supplementary file 1 [file ijms-20-03226-s001.zip › supplementary_Files/Supplemental_Figures_tiff/Figure S4.tif]
